# Integrative genome‐wide chromatin accessibility and transcriptome profiling of diffuse large B‐cell lymphoma

**DOI:** 10.1002/ctm2.975

**Published:** 2022-07-20

**Authors:** Ying Fang, Mu‐Chen Zhang, Peng‐Peng Xu, Su‐Jiang Zhang, Li Wang, Shu Cheng, Di Fu, Chun‐Kang Chang, Xiao‐Jian Sun, Yan Zhao, Yi‐Jia Tang, Xin Tian, Hong‐Mei Yi, Feng Liu, Wei‐Li Zhao

**Affiliations:** ^1^ Shanghai Institute of Hematology, State Key Laboratory of Medical Genomics, National Research Center for Translational Medicine Rui Jin Hospital Affiliated to Shanghai Jiao Tong University School of Medicine Shanghai China; ^2^ Pôle de Recherches Sino‐Français en Science du Vivant et Génomique Laboratory of Molecular Pathology, Shanghai 200025, China; U1165 Inserm/Université Paris 7, Hôpital Saint Louis Paris France; ^3^ Department of Hematology Shanghai Jiao Tong University Affiliated Sixth People's Hospital Shanghai China; ^4^ Department of Pathology Shanghai Rui Jin Hospital; Shanghai Jiao Tong University School of Medicine Shanghai China; ^5^ Department of Pathology, Shanghai Rui Jin Hospital Shanghai Jiao Tong University School of Medicine Shanghai China

Dear Editor,

Diffuse large B‐cell lymphoma (DLBCL) represents the most common neoplastic disorder of B‐lymphocytes with significant clinicopathological, immunophenotypic and molecular heterogeneity.^1^ Aiming to investigate the epigenomic and transcriptomic signatures as well as the underlying pathogenetic mechanism and therapeutic rationale of DLBCL, we performed assay for transposase‐accessible chromatin using sequencing (ATAC‐seq) on 10 DLBCL patient–derived xenograft (PDX) models and identified 93 221 reproducible accessible peaks per PDX (Figures S1–S4). In an unsupervised clustering analysis with previously published ATAC‐seq data on major haematological cell types,^2^, ^3^ all PDX tumours clustered together and, as expected, their closest normal cell types were cells of the B lymphocyte lineage, including naïve B (CD19^pos^CD27^neg^), memory B (CD19^pos^CD27^pos^), plasmablast (CD19^pos^CD27^pos^CD138^pos^CD20^neg^) and common lymphoid progenitors (CLP, Lin^neg^CD34^pos^CD38^pos^CD10^pos^CD45RA^pos^) (Figures 1A and S5a). Using the chromatin accessibility profiles of normal B cells for comparison, we identified a set of differentially accessible regions (DARs, Wald test, *p* adj < .01 by pair‐wise comparison) specifically active in PDX tumours (Figure 1B, cluster 1; Figure S5b). The PDX‐specific DARs were significantly associated with leukocyte activation, cytokine‐mediated signalling, I‐kappa‐B/NF‐kappa‐B (NF‐κB) signalling and MAP kinase activity according to GREAT analysis (see the Supporting Information section) (Figure 1C). Moreover, chromVAR analysis (see the Supporting Information section) of the ATAC‐seq data revealed a group of transcription factor (TF)‐binding motifs highly enriched in PDX tumours, including the binding sequence motifs for AP‐1 family, NF‐κB family and POU family (Figure 1D–F).

**FIGURE 1:**
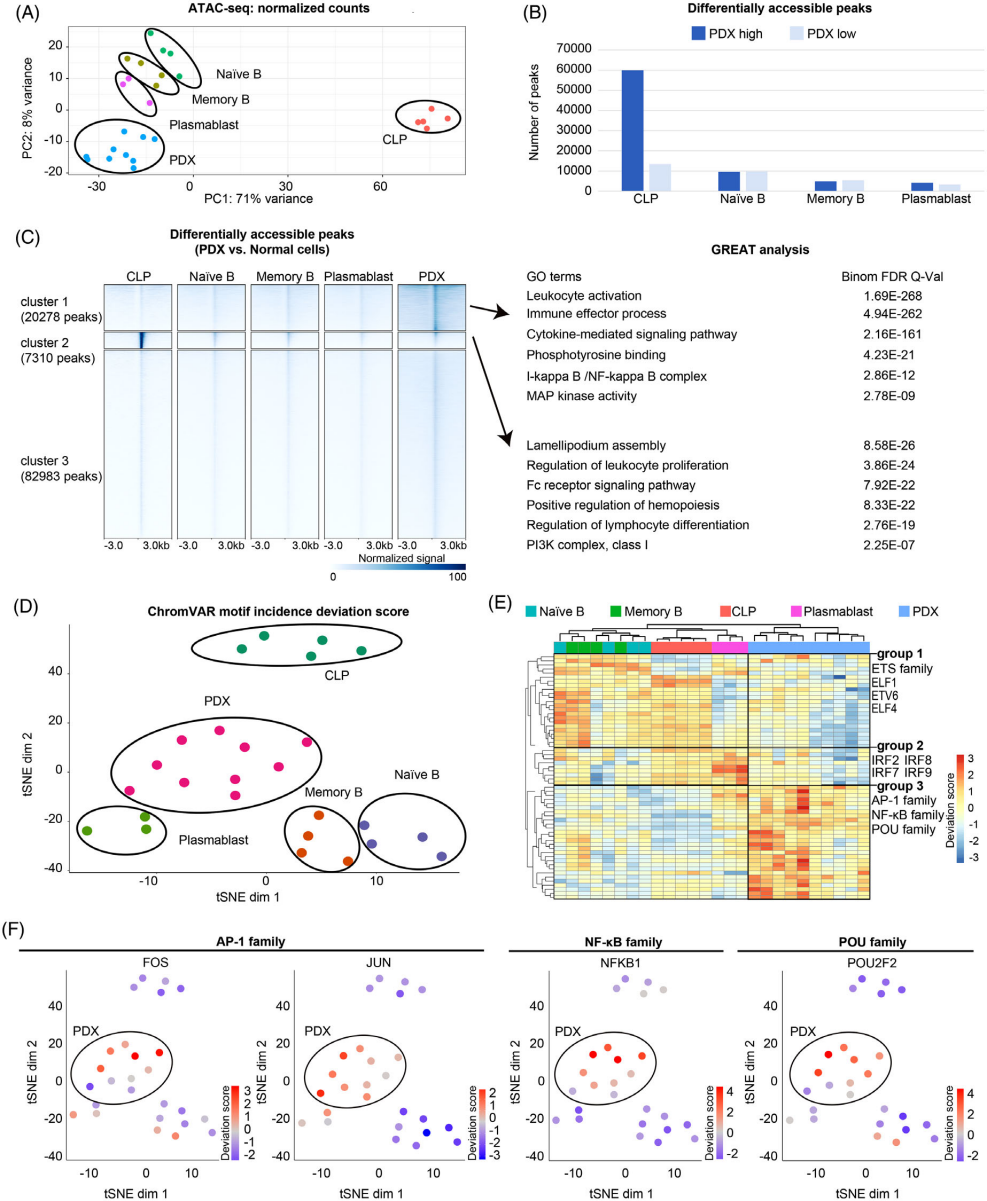
*Cis*‐regulatory signatures of diffuse large B‐cell lymphoma (DLBCL) patient‐derived xenograft (PDX) models. (A) Principal component analysis (PCA) plot of normalized reads counts in assay for transposase‐accessible chromatin using sequencing (ATAC‐seq) peaks. Common lymphoid progenitors (CLP) (*n* = 5), naïve B (*n* = 4), memory B (*n* = 4), plasmablast (*n* = 3) and PDX models (*n* = 10). (B) Differentially accessible peaks between PDX models and each normal cell type. Dark blue shows the peaks that are more accessible in PDX models. Light blue shows the peaks that are less accessible in PDX models. (C) Density heat map of normalized ATAC‐seq signals. Gene ontology terms associated with these peaks by GREAT analysis are shown on the right. Three clusters of peaks showed sample‐specific accessibility profiles. Cluster 1 and cluster 2 peaks were specific to PDX and CLP, respectively. (D) T‐SNE plot using chromVAR motif incidence deviation scores of the ATAC‐seq experiments. (E) Hierarchical clustering of chromVAR motif deviation scores. *k*‐Means analysis (*k* = 3) revealed many transcription factor (TF) motifs that were respectively more active in differentiating B cells (including CLP, naïve B and memory B; group 1), plasmablast (group 2) and PDX (group 3). Representative TF families are shown on the right. (F) T‐SNE plots of representative motif deviation scores enriched in PDX models

Next, to explore the *trans*‐acting factors activated in DLBCL, we performed RNA‐sequencing (RNA‐seq) on PDX samples (*n* = 19, 9/10 PDXs had replicates) and compared them with RNA‐seq data set of CLP, naïve B, memory B and plasmablast,^3^ as well as six reactive lymph node hyperplasia (RLH), a benign condition characterized by the proliferation of non‐neoplastic B lymphocytes (Figures 2A and S5c,d). Based on *k*‐means clustering analysis, we identified two groups of TFs highly up‐regulated in PDX cells as compared to normal B‐cell types (Figure 2B, group 1 and 2 TFs). Group 1 TFs were also highly expressed in RLH and enriched with genes of NF‐κB family (e.g. NFKB1, RELA, RELB), Sp1 zinc‐finger protein family (e.g. KLF16) and MYC/MAX family (e.g. MAX). Group 2 TFs appeared to be specific to PDX tumours and included TFs of AP‐1 family (e.g. BATF, JDP2, FOS, JUN, ATF3, ATF5) and POU family (e.g. POU2F2, POU3F1, POU5F1). By contrast, several well‐established B‐lineage determining TFs (e.g. GATA3, FOXP3 and EOMES), and members of ETS family (e.g. EBF1, ELF1, ETV6),^4^, ^5^, ^6^ were down‐regulated and might contribute to the differentiation defect of PDX and RLH cells (Figure 2B, group 3 and 4 TFs). These results were consistent with gene ontology analysis using all differentially expressed genes (DEGs) (PDX vs. normal cells, or RLH vs. normal cells), which indicated that DEGs enriched in PDX tumours were strongly associated with MAPK pathway that utilizes AP‐1 families of TFs for signal transduction^7^ (Figure 2C). Consistently, the expressions of AP‐1 family and POU family TFs were specifically increased in PDX tumours (Figure S6a,b). Together, these results revealed an aberrant gene expression programme characterized by activated AP‐1 family and POU family TFs, which distinguished DLBCL PDX cells from those of normal B lymphocyte lineage and RLH.

**FIGURE 2:**
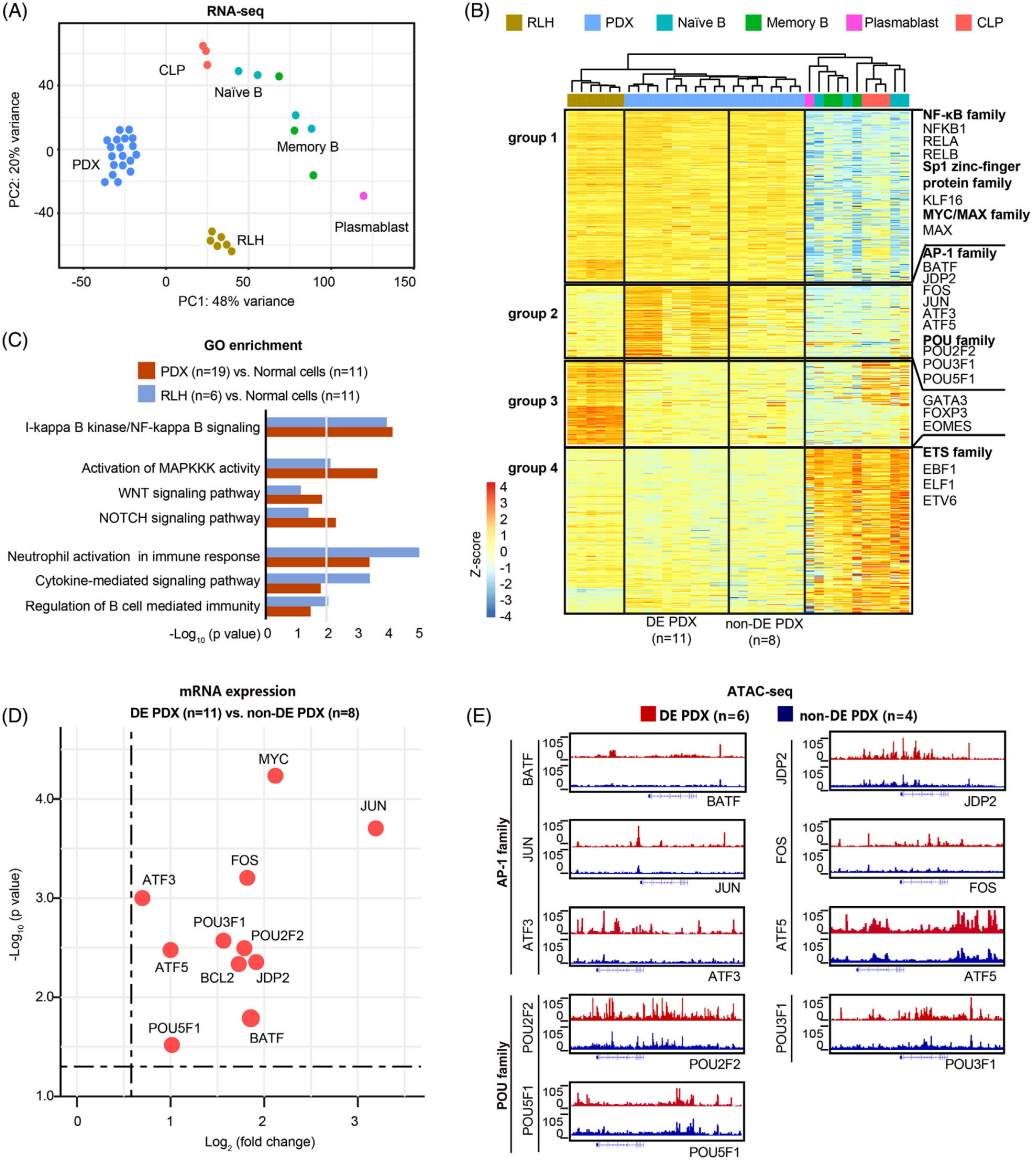
Transcriptomic signatures of diffuse large B‐cell lymphoma (DLBCL) patient‐derived xenograft (PDX) models. (A) Principal component analysis (PCA) plot of RNA‐sequencing (RNA‐seq) data of common lymphoid progenitors (CLP) (*n* = 3), naïve B (*n* = 4), memory B (*n* = 3), plasmablast (*n* = 1), PDX models (9/10 PDXs had replicates, *n* = 19) and reactive lymph node hyperplasia (RLH) (*n* = 6). (B) *k*‐Means clustering (*k* = 4) of differentially expressed transcription factors (TFs). Group 1 and 2 TFs were up‐regulated, whereas group 3 and 4 TFs were down‐regulated, in double expressor (DE) subtype and non‐DE subtype PDX cells with respect to cells of the B lymphocyte lineage. (C) Gene ontology terms associated with genes enriched in PDX models (9/10 PDXs had replicates, *n* = 19) or RLH (*n* = 6), as compared to normal cells (*n* = 11), including CLP (*n* = 3), naïve B (*n* = 4), memory B (*n* = 3) and plasmablast (*n* = 1). The line is corresponding to *p* = .01. (D) Dot plot of the differentially expressed genes between DE subtype (5/6 PDX had replicates, *n* = 11) and non‐DE subtype (4/4 PDX had replicates, *n* = 8) of PDX models. (E) Integrative Genome Browser view of assay for transposase‐accessible chromatin using sequencing (ATAC‐seq) peaks in DE subtype (*n* = 6) and non‐DE subtype (*n* = 4) of PDX models at the loci of indicated genes

Of note, we noticed considerable variations of TF expression and binding motif variations between MYC/BCL2 double expressor (DE) and non‐DE subtype of DLBCL PDX. For example, 9 DLBCL‐enriched TFs were highly expressed in DE subtype, including AP‐1 family and POU family (Figures 2D and S7). The chromatin profiles surrounding the loci of these genes also appeared more accessible in DE subtype based on ATAC‐seq data (Figure 2E). We thus hypothesized that these gene regulatory signatures might be associated with the prognosis of these two subtypes of DLBCL. To test this, we examined RNA‐seq data of 186 tumour samples from newly diagnosed DLBCL patients treated with standard immunochemotherapy rituximab, cyclophosphamide, doxorubicin, vincristine and prednisone (R‐CHOP, *n* = 155) or those from a translational clinical trial of tucidinostat plus R‐CHOP (CR‐CHOP; NCT02753647, *n* = 31, Table S1). Of the TFs up‐regulated in DLBCL PDX cells, three – BATF, JDP2 and ATF5 – were significantly up‐regulated in DE subtype under the Bonferroni correction (Figure 3A), and JDP2 was related to the double expression of MYC and BCL2 in DLBCL patients (Figure 3B–E). Univariate analysis showed that sample enrichment scores^8^ of AP‐1 family TFs were significantly related to poor progression‐free survival (Figure 3F,G), the expression of JDP2 alone was prognostically significant in 155 patients received R‐CHOP (Figure 3H,I). Indeed, the Lasso regression analysis showed that, among the AP‐1 family TFs, JDP2 emerged as an independent prognostic factor in R‐CHOP‐treated patients (Figure 3J,K). Additionally, in vitro experiments using a panel of DLBCL cell lines indicated JDP2 as a critical factor in promoting MYC and BCL2 expression (Figure S8), which supported the importance of the AP‐1 TF member, JDP2, in predicting the prognosis of DLBCL patients.

**FIGURE 3:**
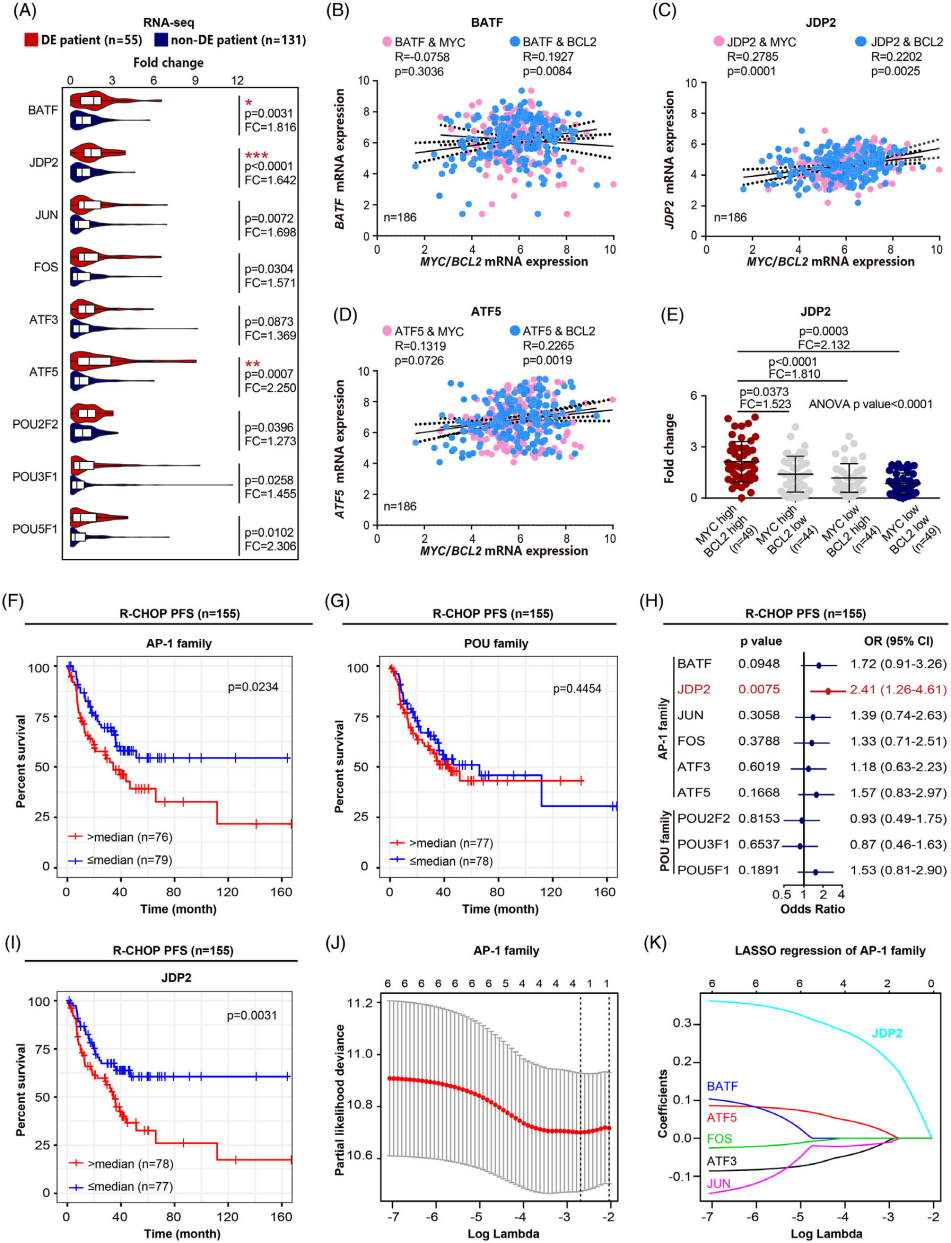
Patient‐derived xenograft (PDX) signatures distinguished double expressor (DE)‐subtype from non‐DE‐subtype correlated with disease progression in diffuse large B‐cell lymphoma (DLBCL) patients. (A) Comparison of the mRNA expression of transcription factors (TFs) between DE subtype (*n* = 55) and non‐DE subtype of DLBCL patients (*n* = 131). Bonferroni correction is applied for multiple testings; * indicates Bonferroni‐adjusted *p* < .05, **p < .01, **p < .001. FC, fold change. (B–D) Pearson correlation analysis of BATF, JDP2 and ATF5 with MYC/BCL2 mRNA expression. (E) Comparison of the mRNA expression of JDP2 among indicated groups. Data are represented as mean ± SD. FC, fold change. (F) Progression‐free survival (PFS) curves of patients treated with R‐CHOP (*n* = 155) according to the expression of AP‐1 family TFs. (G) PFS curves of patients treated with R‐CHOP (*n* = 155) according to the expression of POU family TFs. (H) Kaplan–Meier analysis of patients treated with R‐CHOP (*n* = 155) according to the expression of each DLBCL signature TF. (I) PFS curves of patients treated with R‐CHOP (*n* = 155) according to the expression of JDP2. (J and K) Lasso regression analysis of AP‐1 family TFs

Histone deacetylase inhibitors (HDACIs) are a major group of anti‐cancer agents that act through both epigenetic and non‐epigenetic mechanisms, leading to tumour cell death, apoptosis and cell‐cycle arrest.^9^ In our translational clinical trial (NCT02753647), we found that incorporating HDACI tucidinostat to R‐CHOP (CR‐CHOP) improved the clinical outcome of newly diagnosed DLBCL patients, particularly in DE subtypes that are notoriously refractory to R‐CHOP.^10^ Interestingly, the combined treatment of tucidinostat (12.5 mg/kg/day) and doxorubicin (.6 mg/kg twice a week) profoundly inhibited the growth of PDX tumours, an effect that was more obvious than tucidinostat or doxorubicin alone (Figures 4A,B and S9). We then collected biopsied tumour samples from these PDX models at days 7 and 14 for RNA‐seq analysis. Gene set enrichment analysis revealed that NF‐κB, MAPK, WNT and NOTCH signalling pathways were significantly inhibited upon combined treatment (Figure 4C–F), along with the decreased expression of AP‐1 family TFs (Figure 4G,H). Consistently, the adverse prognostic effect of AP‐1 family TFs, including JDP2, on R‐CHOP‐treated patients was no longer significant among 31 patients received CR‐CHOP (Figure 4I,J).

**FIGURE 4:**
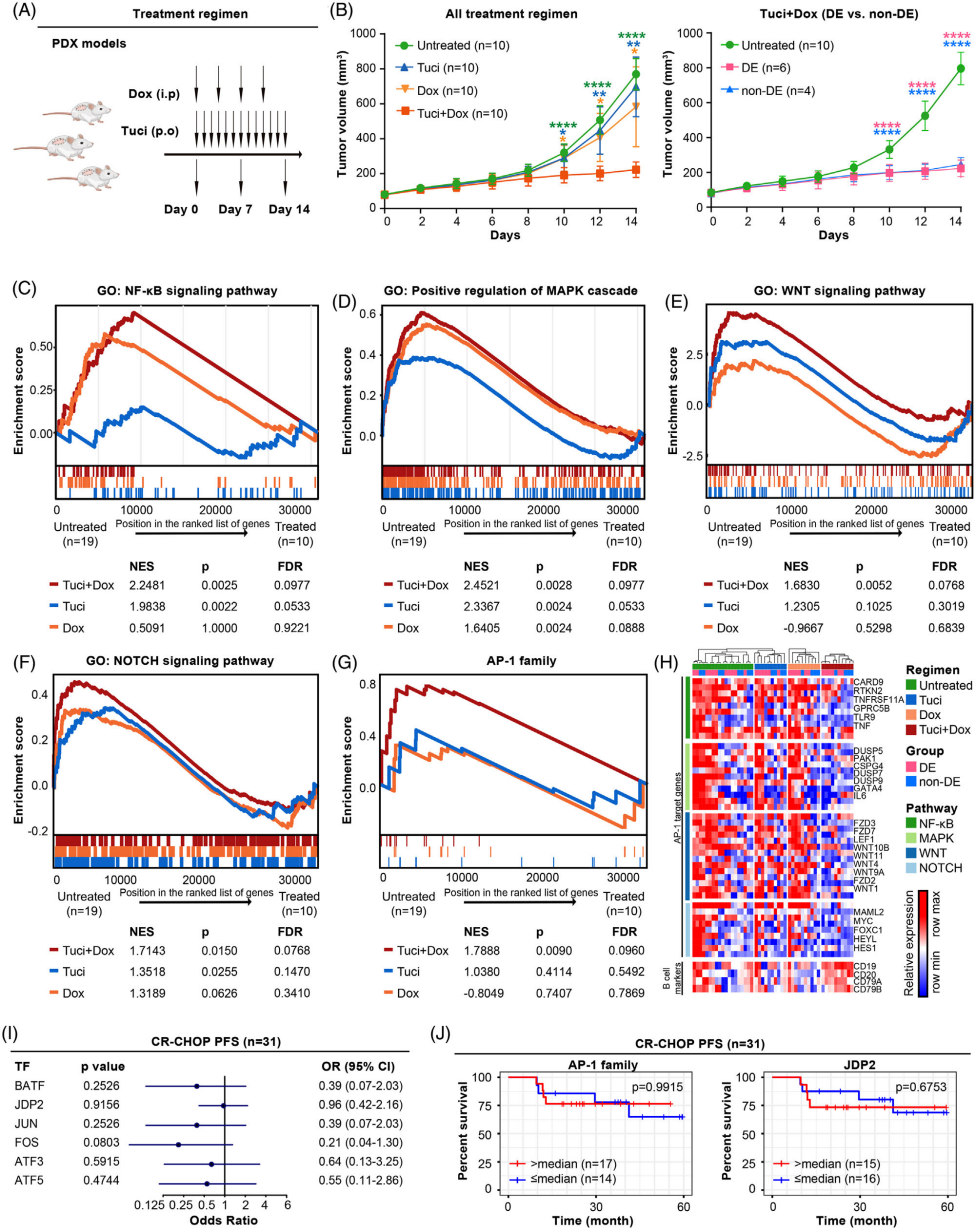
(A) Response of regulatory networks to tucidinostat and/or doxorubicin treatment in diffuse large B‐cell lymphoma (DLBCL). Schematic outline of study design on DLBCL patient‐derived xenograft (PDX) models under tucidinostat and/or doxorubicin treatments. (B) Tumour volume curves of PDX models under tucidinostat and/or doxorubicin treatment (left panel, *n* = 10 each group). *p < .05, **p < .01, ****p < .0001 compared with the untreated (green), tucidinostat (blue) or doxorubicin (yellow) group. Tumour volume curves of double expressor (DE) subtype (pink, *n* = 6) and non‐DE subtype (blue, *n* = 4) of PDX models under tucidinostat and doxorubicin combined treatment (right panel). ****p < .0001 compared with the untreated group. (C–F) Comparison of gene set enrichment analysis between untreated group (*n* = 19, 9/10 PDXs had replicates) and tucidinostat and/or doxorubicin treatment group (*n* = 10), including NF‐κB signalling pathway (C), positive regulation of MAPK cascade (D), WNT signalling pathway (E) and NOTCH signalling pathway (F). NES, normalized enrichment score. (G) Comparison of gene set enrichment analysis of AP‐1 family transcription factors (TFs) between untreated group (*n* = 19, 9/10 PDXs had replicates) and tucidinostat and/or doxorubicin treatment group (*n* = 10). (H) Gene expression profiling of AP‐1 family TFs‐targeted genes upon treatment with tucidinostat and/or doxorubicin. (I) Progression‐free survival (PFS) curves of patients treated with CR‐CHOP (*n* = 31) according to the expression of AP‐1 family TFs. (J) PFS curves of patients treated with CR‐CHOP (*n* = 31) according to the expression of AP‐1 family TFs and JDP2

In conclusion, we established a series of PDX models for studying *cis*‐ and *trans*‐regulatory factors involved in DLBCL tumourigenesis. Together with the analysis of patients receiving standard R‐CHOP or novel CR‐CHOP regimen, our findings provided insights into epigenetic and genetic regulatory signatures driving DLBCL progression and supported the use of tucidinostat in conjunction SI with immunochemotherapy in treating DLBCL with MYC/BCL2 double expression.

## CONFLICT OF INTEREST

The authors declare that there is no conflict of interest that could be perceived as prejudicing the impartiality of the research reported.
