# Supplementary material for: Integrative genome‐wide chromatin accessibility and transcriptome profiling of diffuse large B‐cell lymphoma
Source: Clin Transl Med. 2022 Jul 20;12(7):e975. doi: 10.1002/ctm2.975 (PMC9299574; doi:10.1002/ctm2.975)
Supplement: Supplementary file 1 [file CTM2-12-e975-s001.doc]

# Integrative genome-wide chromatin accessibility and transcriptome profiling of diffuse large B-cell lymphoma

**Running title**: Genomic regulatory signatures of DLBCL

**Authors**

Ying Fang^1,3†^, Mu-Chen Zhang^1†^, Peng-Peng Xu^1†^, Su-Jiang Zhang^1†^, Li Wang^1,2^, Shu Cheng^1^, Di Fu^1^, Chun-Kang Chang^3^, Xiao-Jian Sun^1^, Yan Zhao^1^, Yi-Jia Tang^1^, Xin Tian^1^, Hong-Mei Yi^4^, Feng Liu^1^***** and Wei-Li Zhao^1,2^*****

**Affiliations**

^1^ Shanghai Institute of Hematology, State Key Laboratory of Medical Genomics, National Research Center for Translational Medicine at Shanghai, Rui Jin Hospital Affiliated to Shanghai Jiao Tong University School of Medicine, Shanghai, China.

^2^ Pôle de Recherches Sino-Français en Science du Vivant et Génomique, Laboratory of Molecular Pathology, Shanghai 200025, China; U1165 Inserm/Université Paris 7, Hôpital Saint Louis, Paris, France.

^3^ Department of Hematology, Shanghai Jiao Tong University Affiliated Sixth People’s Hospital, Shanghai, China.

^4^ Department of Pathology, Shanghai Rui Jin Hospital; Shanghai Jiao Tong University School of Medicine, Shanghai, China.

# Supplementary Materials and Methods

## Patients

A total of 186 patients with newly diagnosed DLBCL were enrolled, including 155 patients treated with R-CHOP and 31 patients treated with CR-CHOP (NCT02753647). Histological diagnosis was established according to WHO classification ^1^. Clinical characteristics of the patients were listed in Supplementary Table S1. The study was approved by Shanghai Rui Jin Hospital Review Board and written informed consent was obtained from patients in accordance with the Declaration of Helsinki.

## Cell lines

DLBCL cell lines (OCI-LY-10, Farage, DB, OCI-LY-7, and SU-DHL-4) were obtained from the American Type Culture Collection. Cells were grown in RPMI-1640 (Farage, DB, and SU-DHL-4) or Iscove's MDM (OCI-LY-7 and OCI-LY-10), supplemented with 10% heat-inactivated fetal bovine serum in a humidified atmosphere containing 5% CO_2_ at 37°C.

## Establishment of PDX models

Four-week-old female NOD/LtSz-scid IL2 receptor γ^−/−^ NSG and NOD-SCID mice were obtained from Shanghai Laboratory Animal Center (Shanghai, China) for the establishment and maintenance of PDX models. Ketamine/xylazine cocktail at a ratio of 100 mg/kg:20 mg/kg was used to anesthetize mice. Heterotopic PDX models were generated as previously described ^2^. Tumor samples of DLBCL were collected by ultrasound-guided core needle biopsy and stored in 4°C Hanks balanced salt solution supplemented with antibiotics. Each fresh tumor sample was cut into ~3 mm^3^ pieces and kept in sterile Hanks balanced salt solution until xenotransplantation. Three to six female NSG mice were subcutaneously implanted into the dorsal region with primary tumor sample. After xenotransplantation, animals were evaluated regularly and euthanized when tumors exceeded 12 mm in any dimension. Heterotopic PDX models were maintained by passaging tumor tissue directly from NSG to NOD-SCID mice. Among all tumor samples of 186 newly diagnosed DLBCL patients, extra tissues of 83 tumors were used to generate PDX models. Successful xenograft growth was established from 16 tissue samples (P0, success rate 19.3%), while attempts with the other 67 tissue samples failed (Supplementary Figure S1a). The success rate of establishing DLBCL PDX models is consistent with previous studies ^3^.

To determine whether certain clinical characteristics correlated with the efficiency of PDX model establishment. Clinical characteristic of the patients giving rise to PDX models (n=16) were compared to those failed in PDX model establishment (n=67). These two groups are largely comparable by most clinical characteristics (e.g., gender, age, Ann Arbor stage), except that those with xenografting success showed a higher incidence of DE subtype of DLBCL, characterized by co-expression of MYC and BCL2 oncoprotein, than failed models (56.3% vs. 17.9%, p=0.0033) (Supplementary Figure S1b). Consistently, RNA-seq analysis indicated that 1039 genes significantly up-regulated (fold change>1.5, p<0.05) in tumor cells giving rise to PDX models were strongly associated with DNA replication (Supplementary Figure S1c-d). Once the initial xenografts grew into sizable tumors, tumor tissues were dissected and serially passaged in NOD-SCID mice for maintenance. From 16 starting PDX models (P0), 10 were able to be passaged for 3-5 generations (P3-P5) during this study, including 6 that were initially characterized as DE subtype and 4 as non-DE subtype of DLBCL (Supplementary Figure S1e, Supplementary Figure S2). Pearson correlation analysis of mRNA expression suggested that PDX models preserved the genetic integrity of parental tumors (Supplementary Figure S3a-e). This was further supported by a panel of DLBCL markers (Ki67, CD20, CD79, CD35, CD10, BCL6, MUM1, BCL2, and MYC), as well as morphological and immunohistochemical features (Supplementary Figure S3f-g).

## Therapeutic regimen of PDX models

Tucidinostat and doxorubicin, either alone or in combination, were applied to PDX models with a low passage number (P3-P5) to preserve the genetic integrity of the parental tumors. Tumor volumes were calculated as 0.5×a×b^2^, where ‘a’ is the length and ‘b’ is the width. Treatments were started after the tumor became about 0.5×0.5 cm on surface (day 0). The dose and administration schedule were as follows: doxorubicin 0.6 mg/kg twice a week, tucidinostat 12.5 mg/kg/day for two weeks while the control group was untreated, as previously described ^5,6^. Tumor-bearing mice were then euthanized by CO_2_ asphyxiation. Animals were used according to the ARRIVE guidelines and the protocols approved by Shanghai Rui Jin Hospital Animal Care and Use Committee.

## Immunohistochemistry

Immunohistochemistry was performed on 5 μm paraffin sections with an indirect immunoperoxidase method using antibodies against Ki67, CD20, CD79, CD35, CD10, BCL6, MUM1, BCL2, MYC (Dako, Glostrup, Denmark). The cell of origin profile was determined by Hans algorithm, with a 30% cut-off value of CD10, BCL6, and MUM1 (Dako, Glostrup, Denmark). The cut-off value of BCL2 and MYC were 50% and 40%, respectively ^1^.

## ATAC-seq

Biopsies of PDX models were obtained by the punctuation of the tumor mass using a modified 14-gauge needle (BARD, Arizona, USA), preserved as small aliquots in BAMBANKER (FUJIFILM Wako Pure Chemical Corporation, Tokyo, Japan), and stored at -80°C. Prior to each experiment, the aliquot was thawed on ice and homogenized using a pestle for about 10 minutes in 1 mL of nuclei isolation buffer (10 mM Tris-HCl pH 7.4, 10 mM NaCl, 3 mM MgCl_2_, 0.1% lgepal, 1×protease inhibitors). After passing through a 40 μm filter, the nuclei suspension was counted with a glass hemocytometer. For each ATAC-seq experiment, 50000 nuclei were used as input according to Corces et al ^7^. Each ATAC-seq library was sequenced by Illumina HiSeq 2000 at the depth of 40-60 million per sample (150 bp paired-end reads). Raw sequencing reads were aligned against the human reference genome (*GRCh37/hg19*) using Bowtie2 (v2.2.9) ^8^ with default settings. PCR duplicates were removed by SAMtools (v1.3.1) ^9^. ATAC-seq fragment length distribution in peaks was examined using ataqv (v1.2.1) ^10^. Normalized bigwig files were generated by deepTools (v3.3.1) using the bamCoverage –binSize 10 –normalizeUsing RPKM ^11^. ATAC-seq peak profiles were visualized with Integrative Genomics Viewer ^12^. Peaks were called by MACS2 (2.1.0.20151222) ^13^ using the following parameters: -f BAM -g hs -q 0.01 --keep-dup all. Peak summits were extended 250 bp in both directions, followed by removal of ENCODE blacklisted regions using BEDTools (v2.29.0) ^14^. Peaks called in both biological replicates of each PDX were retained as reproducible peaks. Distal ATAC-seq peaks were those that did not overlap with regions at transcription start sites+/-100bp (hg19 reference genes). The same ATAC-seq data processing pipeline was also used to re-process the ATAC-seq fastq data of normal B cells in published studies ^7,15^. The distal peaks from all samples were merged to generate a master bed file to calculate the reads that overlap with each peak. The resulted reads count table (peaks by samples) was used as input for the R package DESeq2 (v1.32.0) ^16^ for normalization using the vst function and for differential accessibility analysis between samples. Inter-dataset batch effect was removed by the removeBatchEffect function of the R package ‘limma’ (v3.38.3) prior to sample clustering analysis. The peaks with less than 200 reads in all samples were deemed as weak peaks and discarded. Differential peaks were those with adjusted p value <0.05. GO and pathway associated with DARs were annotated by Genomic Regions Enrichment of Annotations Tool (GREAT) ^17^. The peak count table was also used as input for ChromVAR (v1.40.0) analysis using JASPAR TF motif collection ^18^. Quality control of the ATAC-seq data was included in Supplementary Figure S4.

## RNA-seq

Total mRNA was extracted from frozen tumor samples of 186 newly diagnosed DLBCL patients and 10 PDX models (n=19, 9/10 PDXs had replicates) using Trizol (Invitrogen, California, USA) and RNeasy MinElute Cleanup Kit (Qiagen, Dusseldorf, German) according to the manufacturer’s instructions. RNA integrity was assessed using RNA 6000 Nano Kit (Agilent, California, USA) on Agilent 2100 Bioanalyzer (Agilent, California, USA). RNA-seq was performed using Illumina HiSeq 2000 (Illumina, California, USA) at the depth of 20-40 million 150 bp paired-end reads per sample. Raw fastq data were aligned with a human transcriptome index based on Homo_sapiens.GRCh37.cdna.all using kallisto (v0.46.0). The R package ‘limma’ (v3.38.3) was used to normalize raw reads, remove inter-dataset batch effects, and obtain DEGs. R package ‘clusterProfiler’ (v3.10.1) was used for gene set enrichment analysis, GO enrichment analysis, and data visualization.

## Sample enrichment scores (SES)

The sample enrichment scores (SES) were computed by Auto-Compare-SES (https://sites.google.com/site/fredsoftwares/products/autocompare_ses) with normalized settings ^19^.

## Lasso Cox regression

A robust likelihood-based survival modeling was applied to reduce the dimension and obtain robust survival-associated TFs. The Lasso regression was used to identify the TFs with independent prognostic value by the R package “glmnet” ^20^.

## Western blot

Western blot analysis was performed as previously described ^21^. Rabbit anti-MYC antibody (ab32072, Abcam, Cambridge, MA, USA) and rabbit anti-BCL2 antibody (ab32124, Abcam, Cambridge, MA, USA) were used for western blot. An anti-GAPDH antibody (3683S, Cell Signaling Technology, Danvers, MA, USA) was used as the endogenous loading control. Unprocessed original images of western blot were provided in Supplementary information.

## Plasmids, lentivirus construction, and short hairpin RNAs

Full-length *JDP2* was cloned into the vector pLKO5-EGFP for overexpression (Shanghai Xitubio Biotechnology Co., Ltd, Shanghai, China). Short hairpin RNA (shRNA) lentivirus of *JDP2* was constructed by Xitubio Biotech. ShRNA sequences are provided in Supplementary Table S2.

## Quantitative Real-time PCR

## Total mRNA was extracted using TRIzol reagent (Invitrogen, Shanghai, China). Complementary DNA was synthesized using Prime Script RT Reagent Kit with gDNA Eraser (TaKaRa, Dalian, China). qRT-PCR was performed by SYBR Premix Ex TaqTM II (TaKaRa) and ABI ViiA7 (Applied Biosystems, Foster City, CA, USA) with primers against *JDP2*, *JUN*, *FOS*, *ATF3*, *ATF5*, *POU2F2*, *POU3F1*, *POU5F1*, *MYC*, *BCL2*, and *GAPDH* was used as an endogenous control. SU-DHL-4 cells were used for calibration. Relative expressions were calculated by the method of _ΔΔ_CT. The primer sequences of above genes were list in Supplementary Table S3.

## Statistics

All statistical analyses were performed in GraphPad Prism software (GraphPad Software, San Diego, CA, version 7.0), SPSS v23.0 and R v3.6.1 Survival Estimates were calculated using the Kaplan-Meier method and survival curves were compared by the log-rank test. Univariate hazard estimate was generated with Cox proportional hazards models. Unpaired t-test with or without Welch’s correction was used to compare different groups and spearman rank correlation was used to calculate the correlation between two groups. Fisher's exact tests were applied to compare non-ordinal categorical variables. A two-sided p value of <0.05 was considered statistically significant.

## Study approval

The study was approved by Shanghai Rui Jin Hospital Review Board and written informed consent was obtained from patients in accordance with the Declaration of Helsinki. Animals were used according to the ARRIVE guidelines and the protocols approved by Shanghai Rui Jin Hospital Animal Care and Use Committee.

# References

1. Swerdlow SH, Campo E, Pileri SA, et al. The 2016 revision of the World Health Organization classification of lymphoid neoplasms. *Blood.* 2016;127(20):2375-2390.

2. Hidalgo M, Amant F, Biankin AV, et al. Patient-derived xenograft models: an emerging platform for translational cancer research. *Cancer discovery.* 2014;4(9):998-1013.

3. Townsend EC, Murakami MA, Christodoulou A, et al. The Public Repository of Xenografts Enables Discovery and Randomized Phase II-like Trials in Mice. *Cancer cell.* 2016;29(4):574-586.

4. Hu S, Xu-Monette ZY, Tzankov A, et al. MYC/BCL2 protein coexpression contributes to the inferior survival of activated B-cell subtype of diffuse large B-cell lymphoma and demonstrates high-risk gene expression signatures: a report from The International DLBCL Rituximab-CHOP Consortium Program. *Blood.* 2013;121(20):4021-4031; quiz 4250.

5. Clozel T, Yang S, Elstrom RL, et al. Mechanism-based epigenetic chemosensitization therapy of diffuse large B-cell lymphoma. *Cancer discovery.* 2013;3(9):1002-1019.

6. Gong K, Xie J, Yi H, Li W. CS055 (Chidamide/HBI-8000), a novel histone deacetylase inhibitor, induces G1 arrest, ROS-dependent apoptosis and differentiation in human leukaemia cells. *The Biochemical journal.* 2012;443(3):735-746.

7. Corces MR, Buenrostro JD, Wu B, et al. Lineage-specific and single-cell chromatin accessibility charts human hematopoiesis and leukemia evolution. *Nature genetics.* 2016;48(10):1193-1203.

8. Langmead B, Salzberg SL. Fast gapped-read alignment with Bowtie 2. *Nature methods.* 2012;9(4):357-359.

9. Li H, Handsaker B, Wysoker A, et al. The Sequence Alignment/Map format and SAMtools. *Bioinformatics (Oxford, England).* 2009;25(16):2078-2079.

10. Orchard P, Kyono Y, Hensley J, Kitzman JO, Parker SCJ. Quantification, Dynamic Visualization, and Validation of Bias in ATAC-Seq Data with ataqv. *Cell systems.* 2020;10(3):298-306.e294.

11. Bulut-Karslioglu A, De La Rosa-Velázquez IA, Ramirez F, et al. Suv39h-dependent H3K9me3 marks intact retrotransposons and silences LINE elements in mouse embryonic stem cells. *Molecular cell.* 2014;55(2):277-290.

12. Robinson JT, Thorvaldsdóttir H, Winckler W, et al. Integrative genomics viewer. *Nature biotechnology.* 2011;29(1):24-26.

13. Liu T. Use model-based Analysis of ChIP-Seq (MACS) to analyze short reads generated by sequencing protein-DNA interactions in embryonic stem cells. *Methods in molecular biology (Clifton, NJ).* 2014;1150:81-95.

14. Quinlan AR, Hall IM. BEDTools: a flexible suite of utilities for comparing genomic features. *Bioinformatics (Oxford, England).* 2010;26(6):841-842.

15. Calderon D, Nguyen MLT, Mezger A, et al. Landscape of stimulation-responsive chromatin across diverse human immune cells. *Nature genetics.* 2019;51(10):1494-1505.

16. Love MI, Huber W, Anders S. Moderated estimation of fold change and dispersion for RNA-seq data with DESeq2. *Genome biology.* 2014;15(12):550.

17. McLean CY, Bristor D, Hiller M, et al. GREAT improves functional interpretation of cis-regulatory regions. *Nature biotechnology.* 2010;28(5):495-501.

18. Mathelier A, Fornes O, Arenillas DJ, et al. JASPAR 2016: a major expansion and update of the open-access database of transcription factor binding profiles. *Nucleic acids research.* 2016;44(D1):D110-115.

19. Tosolini M, Algans C, Pont F, Ycart B, Fournié JJ. Large-scale microarray profiling reveals four stages of immune escape in non-Hodgkin lymphomas. *Oncoimmunology.* 2016;5(7):e1188246.

20. Friedman J, Hastie T, Tibshirani R. Regularization Paths for Generalized Linear Models via Coordinate Descent. *Journal of statistical software.* 2010;33(1):1-22.

21. Huang YH, Cai K, Xu PP, et al. CREBBP/EP300 mutations promoted tumor progression in diffuse large B-cell lymphoma through altering tumor-associated macrophage polarization via FBXW7-NOTCH-CCL2/CSF1 axis. *Signal transduction and targeted therapy.* 2021;6(1):10.

# Supplementary Figure legends

## Supplementary Figure S1.

(a) Illustration of the protocol of PDX model establishment.

(b) Comparison of the clinical characteristics of the primary DLBCL tumors giving rise to PDX models (P0, n=16) and those who failed to establish PDX model establishment (n=67).

(c) Comparison of the transcriptome profiles between primary DLBCL tumors giving rise to PDX models (P0, n=16) and those who failed to PDX model establishment (n=67).

(d) GO enrichment analysis of upregulated differentially expressed genes (c).

(e) Clinical features of stably maintained PDX models (P3-P5, n=10).

## Supplementary Figure S2.

Tumor growth curve of positive and negative controls of PDX models. Hematoxylin and eosin stain of PDX models.

## Supplementary Figure S3.

(a-e) Pearson correlation analysis of mRNA expression of indicated DLBCL markers between PDX model and corresponding patient.

(f) Comparison of immunohistochemistry intensity of indicated DLBCL markers between representative PDX model and corresponding patient.

(g) Hematoxylin and eosin stain and immunohistochemistry of DLBCL markers on representative PDX model. Original magnification ×100.

## Supplementary Figure S4.

(a) Percentage of ATAC-seq peaks overlapped with human gene promoters (hg19) and the Corces dataset. (b) The fragment length of ATAC-seq data.

(c) Test for read count outliers by Cook’s distance as determined by DESeq2 R package. Y-axis shows the log_10_ values of fitted coefficients for the ATAC-seq peaks in a given sample.

(d-e) ATAC-seq peaks in PDX samples (n=10) and normal cells (naïve B, memory B, plasmablast and CLP) at the loci of indicated genes. Dot plot of the differentially expressed genes between DE subtype (5/6 PDX had replicates, n=11) and non-DE subtype (4/4 PDX had replicates, n=8) of PDX models.

## Supplementary Figure S5.

(a) Hierarchical clustering of deviation score of ChromVAR analysis of ATAC-seq using normal hematopoietic cells and PDX models (n=10). MEP, megakaryocyte-erythroid progenitor; Ery, erythrocyte; CMP, common myeloid progenitor; HSC, hemopoietic stem cell; MPP, multi-potent progenitor; Mono, monocyte; LMPP, lymphoid-primed multi-potent progenitor; GMP, granulocyte macrophage progenitor; CLP, common lymphoid progenitor.

(b) Differentially accessible peaks between PDX models and each normal cell type. Dark blue shows the peaks that are more accessible in PDX models. Light blue shows the peaks that are less accessible in PDX models. Dark orange shows the peaks that are more accessible in CLP. Light orange shows the peaks that are less accessible in CLP.

(c) Principal component analysis (PCA) plot of RNA-seq data of normal hematopoietic cells and PDX models (n=19). CLP, common lymphoid progenitor; CMP, common myeloid progenitor; Ery, erythrocyte; GMP, granulocyte macrophage progenitor; HSC, hemopoietic stem cell; LMPP, lymphoid-primed multi-potent progenitor; MEP, megakaryocyte-erythroid progenitor; Mono, monocyte; MPP, multi-potent progenitor.

(d) B-cell receptor gene expression profiling of RLH (n=6), PDX (9/10 PDXs had replicates, n=19), and normal cells (n=11).

## Supplementary Figure S6.

(a) Volcano plots of select TF families differentially expressed between PDX models (9/10 PDXs had replicates, n=19) and normal cells (n=11).

(b) Volcano plots of select TF families differentially expressed between PDX models (9/10 PDXs had replicates, n=19) and RLH (n=6).

## Supplementary Figure S7.

(a) Overview of chromatin accessibility and mRNA expression of MYC and BCL2 between DE (n=6) and non-DE (n=4) PDX models.

(b) mRNA expression of MYC and BCL2 between DE (n=11) and non-DE (n=8) PDX models.

## Supplementary Figure S8.

(a) MYC and BCL2 expression in B cell lymphoma cell lines. Data are represented as mean ± SD. Assays were set up in triplicate.

(b) Immunostaining of MYC and BCL2 in OCI-LY-10 and Farage cells. The scale bar represents 200 μm.

(c) JDP2, MYC, and BCL2 expression in OCI-LY-10 (left panel) and Farage (right panel) cells transfected with scramble shRNA/*JDP2* shRNA or vector control/*JDP2* assessed by qRT-PCR and western blot. The scramble values were normalized to 1. Assays were set up in triplicate.

(d-e) Unprocessed image in a and c.

## Supplementary Figure S9.

(a) Immunohistochemistry staining CD20 was performed on the biopsied samples of PDX models before and after treatment. The scale bar represents 200 μm.

(b) Gene expression profiling of DLBCL-related genes before and after treatment.

# Supplementary Tables

**Supplementary Table S1. Clinical characteristics of patients with DLBCL (n=186)**

| Characteristics | Enrolled patients  (n=186) | | R-CHOP  (n=155) | CR-CHOP  (n=31) |
| --- | --- | --- | --- | --- |
| Age (years) | |  |  |  |
| Median | | 62 (16-85) | 60 (16-85) | 66 (60-75) |
| Gender | |  |  |  |
| Male | | 104 (55.9%) | 85 (54.8%) | 19 (61.3%) |
| Female | | 82 (44.1%) | 70 (45.2%) | 12 (38.7%) |
| ECOG | |  |  |  |
| 0-1 | | 158 (84.9%) | 133 (85.8%) | 25 (80.6%) |
| 2 | | 28 (15.1%) | 22 (14.2%) | 6 (19.4%) |
| Ann Arbor stage | |  |  |  |
| I-II | | 72 (38.7%) | 67 (43.2%) | 5 (16.1%) |
| III-IV | | 114 (61.3%) | 88 (56.8%) | 26 (83.9%) |
| LDH | |  |  |  |
| Normal | | 63 (33.9%) | 58 (37.4%) | 5 (6.1%) |
| Elevated | | 123 (66.1%) | 97 (62.6%) | 26 (83.9%) |
| Extranodal involvement | | |  |  |
| 0-1 | | 105 (56.5%) | 94 (60.6%) | 11 (35.5%) |
| ≥2 | | 81 (43.5%) | 61 (39.4%) | 20 (64.5%) |
| IPI | |  |  |  |
| 0-1 | | 58 (31.2%) | 58 (37.4%) | 0 (0.0%) |
| 2-5 | | 128 (68.8%) | 97 (62.6%) | 31 (100.0%) |
| Cell of origin | |  |  |  |
| GCB | | 52 (28.0%) | 42 (27.1%) | 10 (32.3%) |
| non-GCB | | 134 (72.0%) | 113 (72.9%) | 21 (67.7%) |
| MYC and BCL2 overexpression | | |  |  |
| Yes | | 55 (29.6%) | 46 (29.7%) | 9 (29.0%) |
| No | | 131 (70.4%) | 109 (70.3%) | 22 (71.0%) |

**Supplementary Table S2**. ShRNA sequences

| shRNA | Sequences (5' to 3') |
| --- | --- |
| *JDP2* shRNA | GAGGAAGAGGAGCGAAGGAAA |
| Scramble shRNA | GCGCGATAGCGCTAATAATTT |

**Supplementary Table S3. Primer sequences used in real-time quantitative reverse transcription-PCR**

| Gene | Forward | Reverse |
| --- | --- | --- |
| *JDP2* | 5′-CCCAGCCCGTGAAAAGTGA-3′ | 5′-CGGTGTCGGTTCAGCATCA-3′ |
| *JUN* | 5′-TCCAAGTGCCGAAAAAGGAAG-3′ | 5′-CGAGTTCTGAGCTTTCAAGGT-3′ |
| *FOS* | 5′-CCGGGGATAGCCTCTCTTACT-3′ | 5′-CCAGGTCCGTGCAGAAGTC-3′ |
| *ATF3* | 5′-CCTCTGCGCTGGAATCAGTC-3′ | 5′-TTCTTTCTCGTCGCCTCTTTTT-3′ |
| *ATF5* | 5′-TGGCTCGTAGACTATGGGAAA-3′ | 5′-ATCAACTCGCTCAGTCATCCA-3′ |
| *POU2F2* | 5′-AGCACACAGACACCGAAAGAA-3′ | 5′-GTTGGGGACACGGAGAATGG-3′ |
| *POU3F1* | 5′-CGCTCTACGGTAACGTGTTCT-3′ | 5′-CCAAGCCGGTGATCTCGTG-3′ |
| *POU5F1* | 5′-CTTGAATCCCGAATGGAAAGGG-3′ | 5′-GTGTATATCCCAGGGTGATCCTC-3′ |
| *MYC* | 5′-GGCTCCTGGCAAAAGGTCA-3′ | 5′-CTGCGTAGTTGTGCTGATGT-3′ |
| *BCL2* | 5′-GGTGGGGTCATGTGTGTGG-3′ | 5′-CGGTTCAGGTACTCAGTCATCC-3′ |
| *GAPDH* | 5′-GGAGCGAGATCCCTCCAAAAT-3′ | 5′-GGCTGTTGTCATACTTCTCATGG-3′ |
