# Supplementary material for: Integrative genome‐wide chromatin accessibility and transcriptome profiling of diffuse large B‐cell lymphoma
Source: Clin Transl Med. 2022 Jul 20;12(7):e975. doi: 10.1002/ctm2.975 (PMC9299574; doi:10.1002/ctm2.975)

Fang et al. Supplementary Figure S1

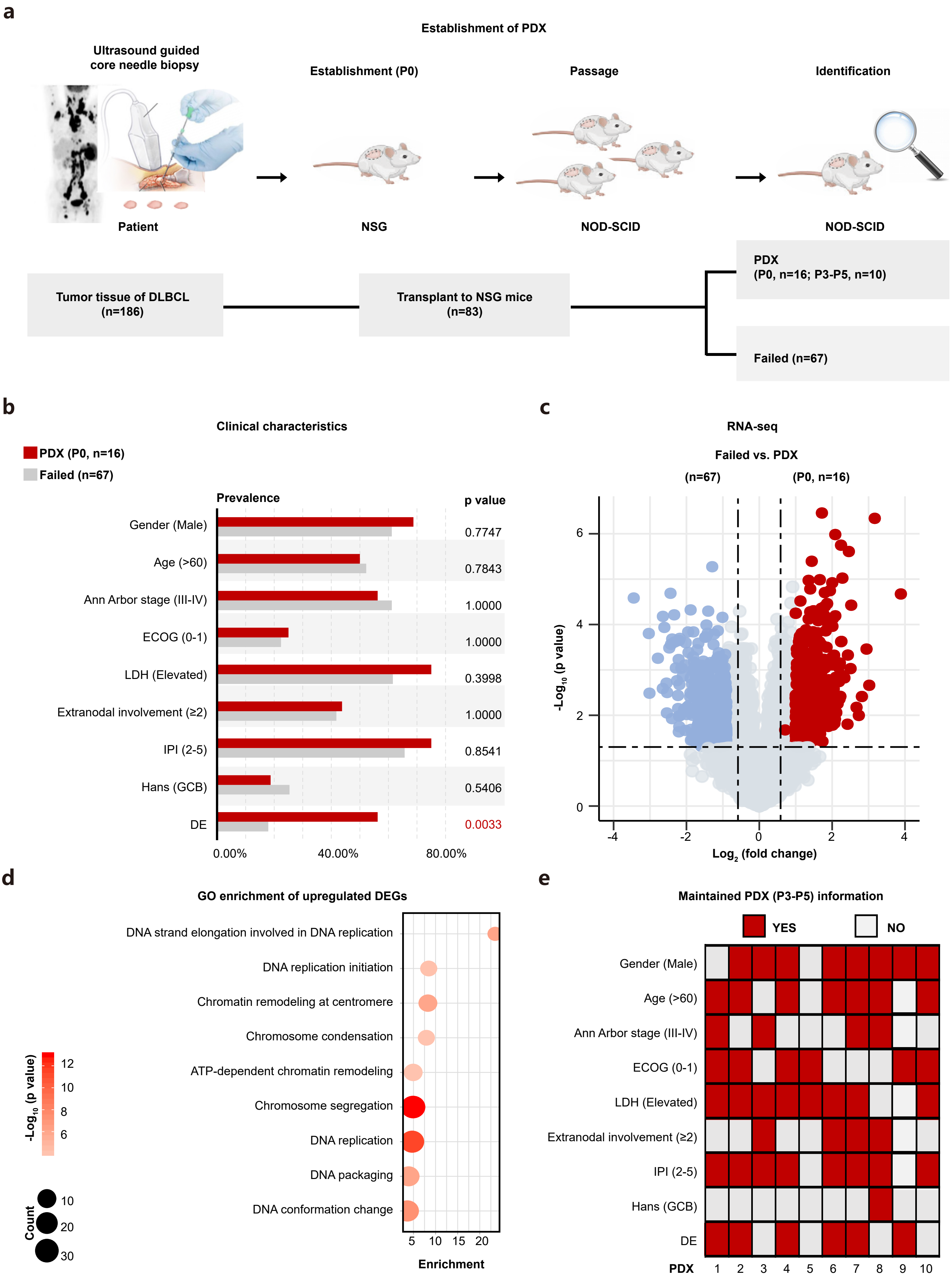

Fang et al. Supplementary Figure S2

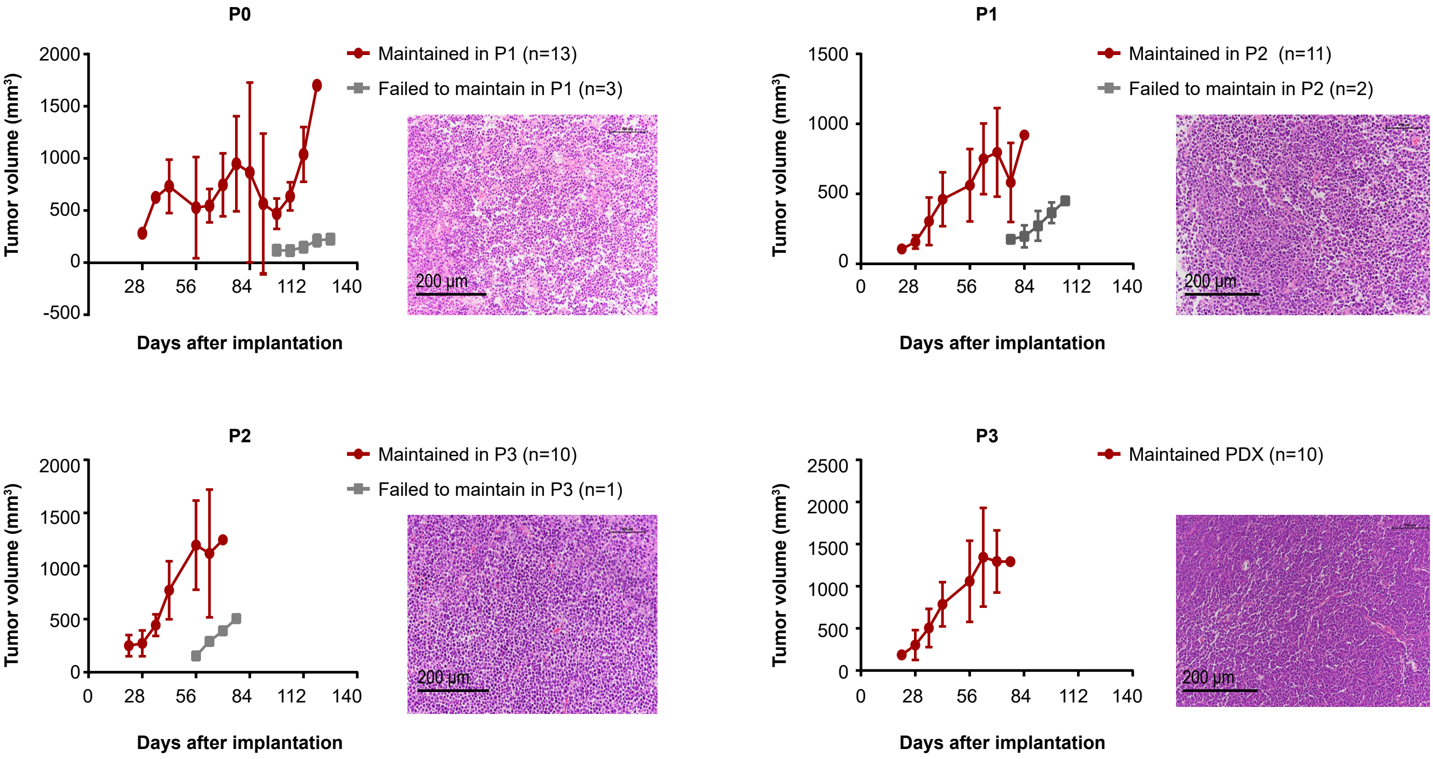

Fang et al. Supplementary Figure S3

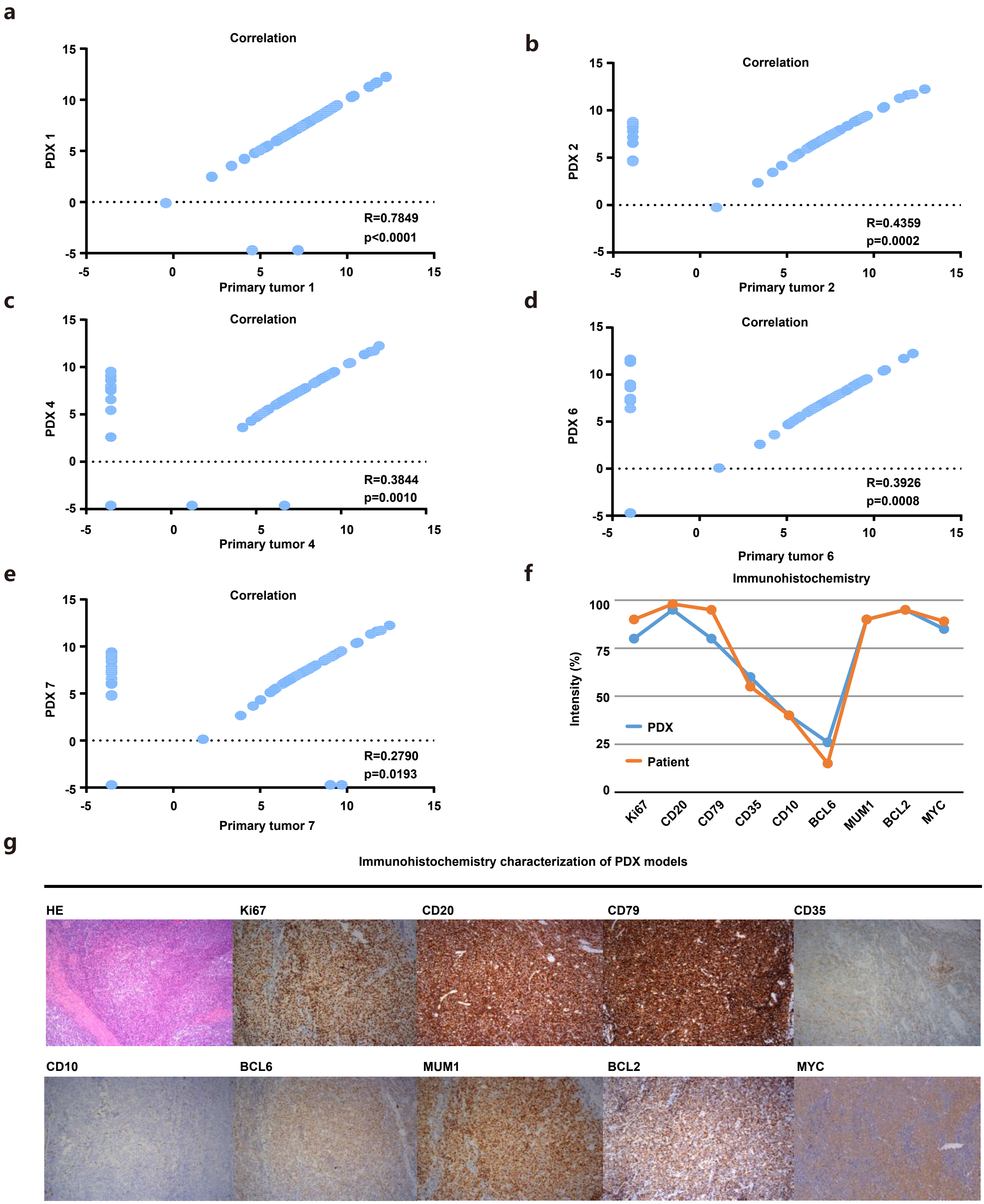

Fang et al. Supplementary Figure S4

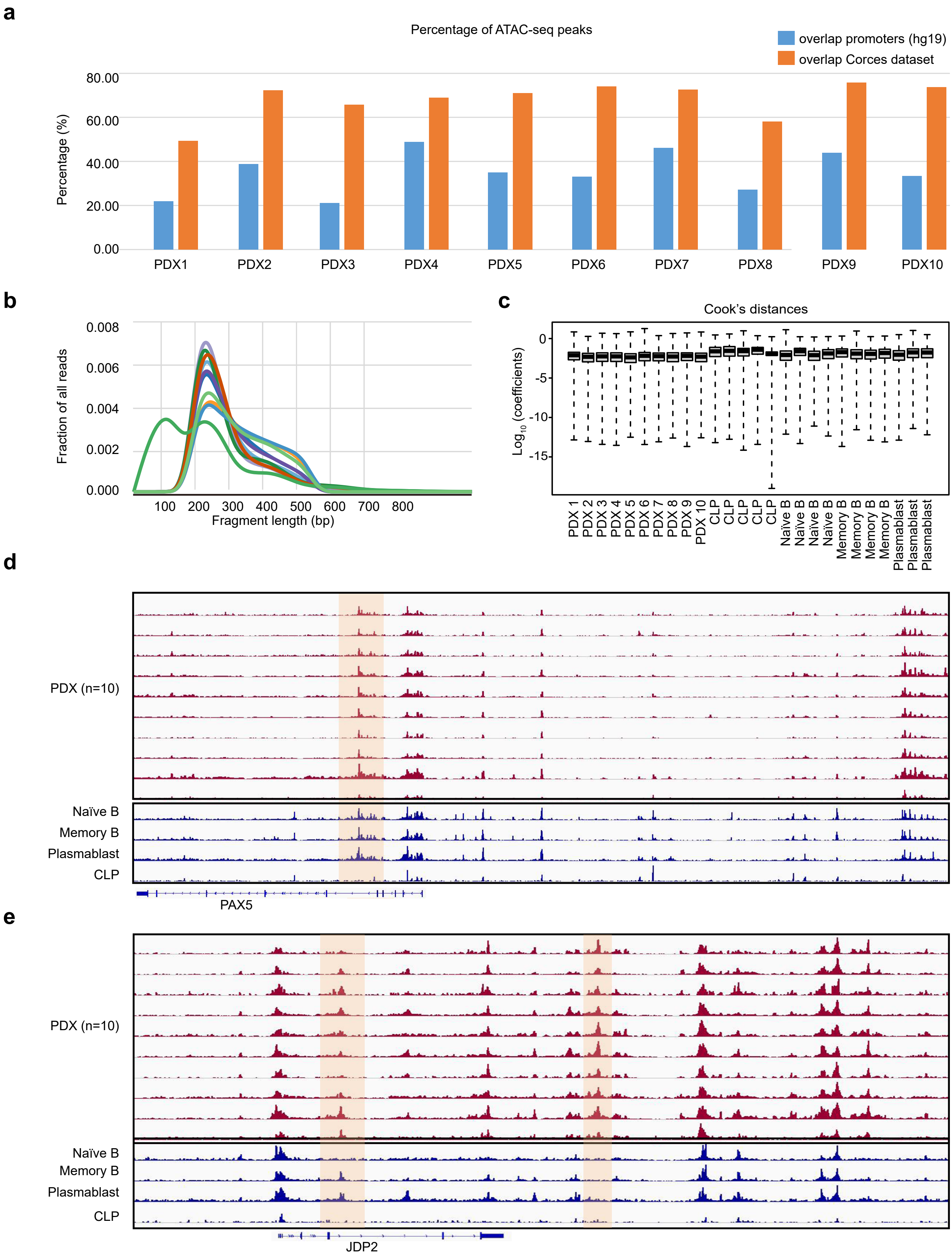

Fang et al. Supplementary Figure S5

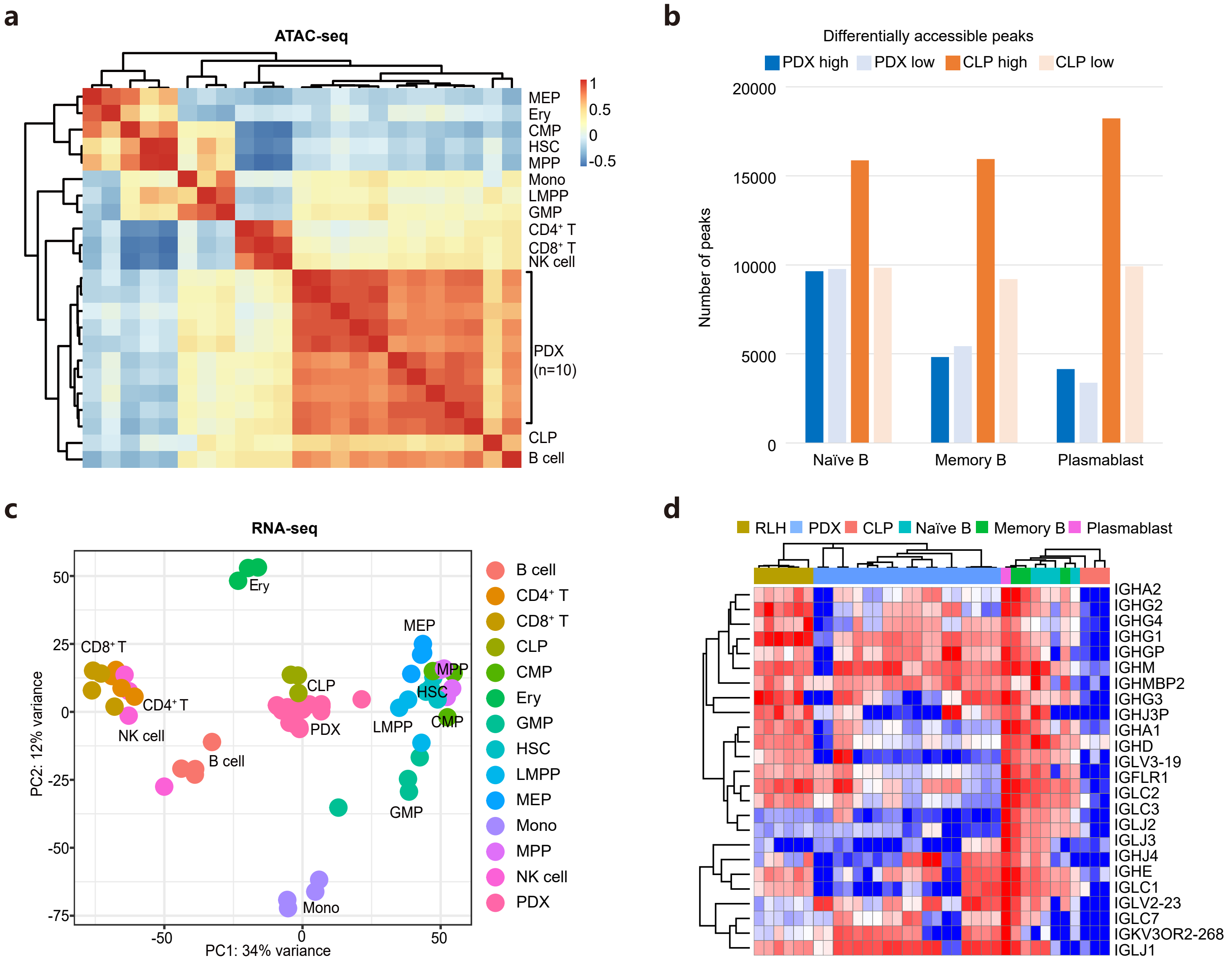

Fang et al. Supplementary Figure S6

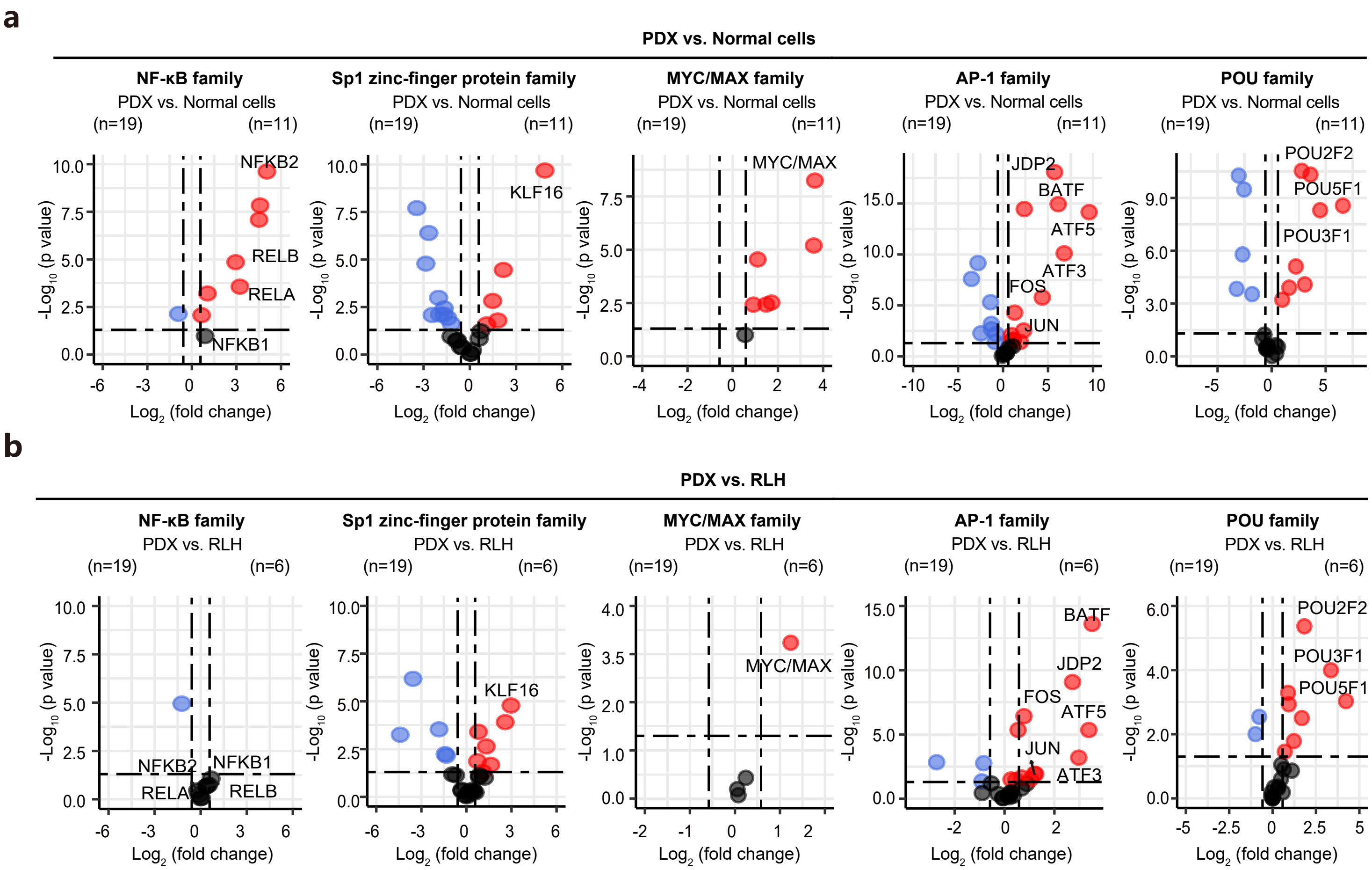

Fang et al. Supplementary Figure S7

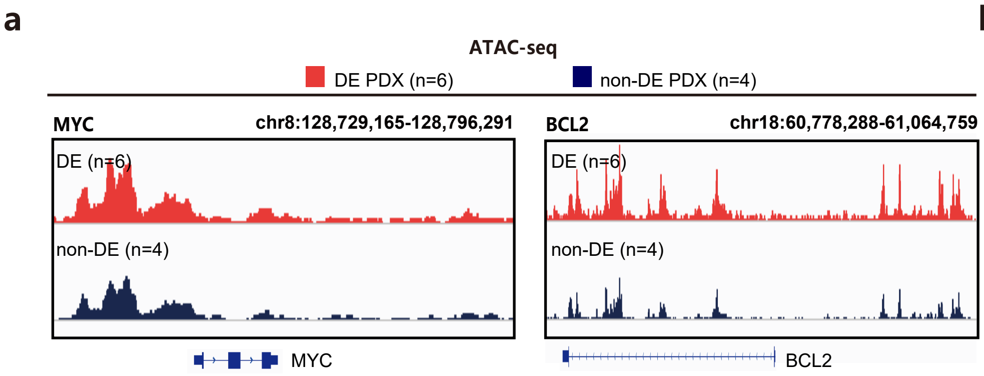

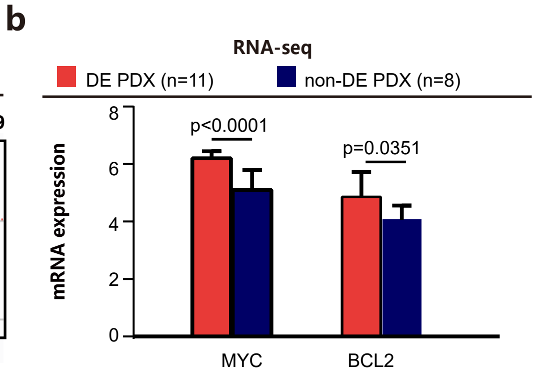

Fang et al. Supplementary Figure S8

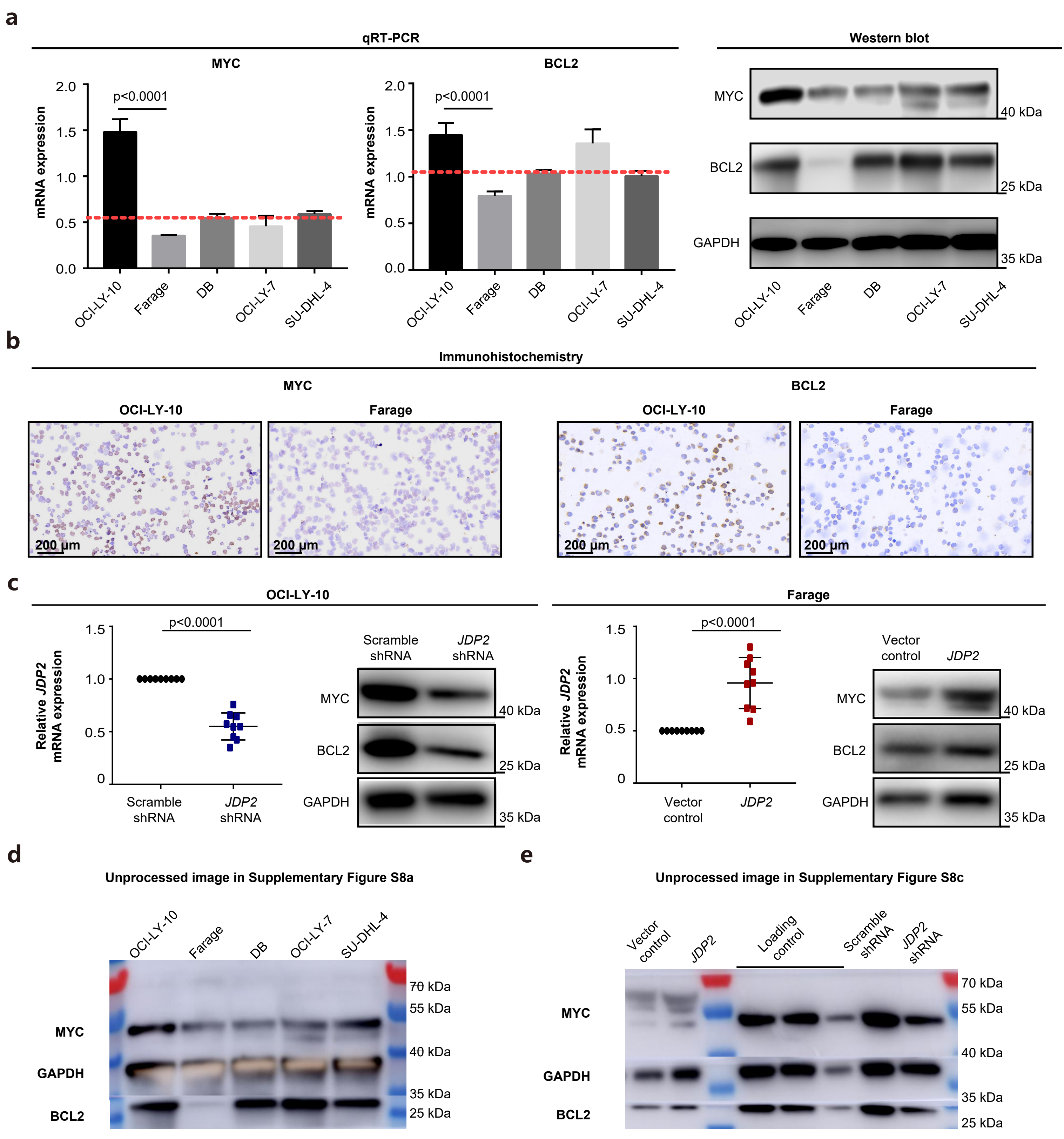

Fang et al. Supplementary Figure S9

a

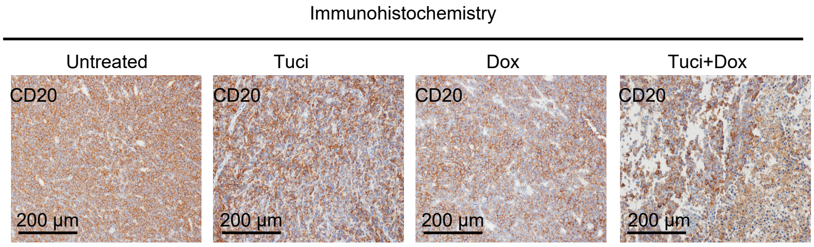

b

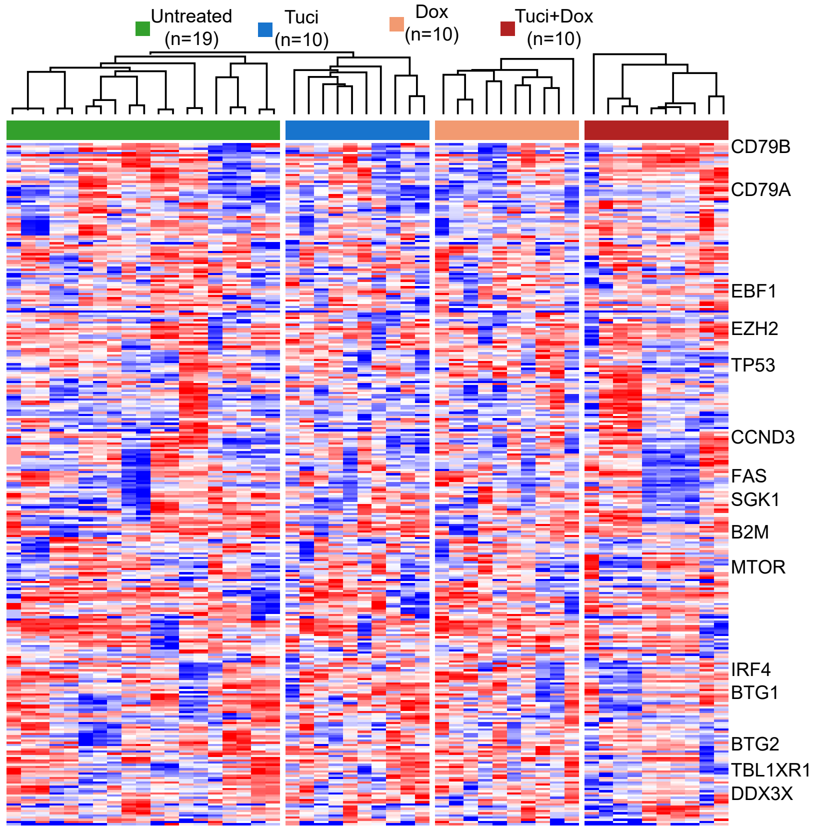

Supplement: Supplementary file 2 [file CTM2-12-e975-s002.pdf]
